# Identification of Clubroot (*Plasmodiophora brassicae*) Resistance Loci in Chinese Cabbage (*Brassica rapa* ssp. *pekinensis*) with Recessive Character

**DOI:** 10.3390/genes15030274

**Published:** 2024-02-22

**Authors:** Hui Zhang, Xitong Liu, Jinyan Zhou, Stephen E. Strelkov, Rudolph Fredua-Agyeman, Shifan Zhang, Fei Li, Guoliang Li, Jian Wu, Rifei Sun, Sheau-Fang Hwang, Shujiang Zhang

**Affiliations:** 1 State Key Laboratory of Vegetable Biobreeding, Institute of Vegetables and Flowers, Chinese Academy of Agricultural Sciences, Beijing 100081, China; zhanghui05@caas.cn (H.Z.); liuxitong102728@163.com (X.L.); zhoujyzjy@163.com (J.Z.); zhangshifan@caas.cn (S.Z.); lifei@caas.cn (F.L.); liguoliang@caas.cn (G.L.); wujian@caas.cn (J.W.); sunrifei@caas.cn (R.S.); 2 Department of Agricultural, Food and Nutritional Science, University of Alberta, Edmonton, AB T6G 2P5, Canada; strelkov@ualberta.ca (S.E.S.); freduaag@ualberta.ca (R.F.-A.)

**Keywords:** *Brassica rapa*, *Plasmodiophora brassicae*, resistance gene, QTL, BSA-seq

## Abstract

The soil-borne pathogen *Plasmodiophora brassicae* is the causal agent of clubroot, a major disease in Chinese cabbage (*Brassica rapa* ssp. *pekinensis*). The host’s resistance genes often confer immunity to only specific pathotypes and may be rapidly overcome. Identification of novel clubroot resistance (CR) from germplasm sources is necessary. In this study, Bap246 was tested by being crossed with different highly susceptible *B. rapa* materials and showed recessive resistance to clubroot. An F_2_ population derived from Bap246 × Bac1344 was used to locate the resistance Quantitative Trait Loci (QTL) by Bulk Segregant Analysis Sequencing (BSA-Seq) and QTL mapping methods. Two QTL on chromosomes A01 (4.67–6.06 Mb) and A08 (10.42–11.43 Mb) were found and named *Cr4Ba1.1* and *Cr4Ba8.1*, respectively. Fifteen and eleven SNP/InDel markers were used to narrow the target regions in the larger F_2_ population to 4.67–5.17 Mb (A01) and 10.70–10.84 Mb (A08), with 85 and 19 candidate genes, respectively. The phenotypic variation explained (PVE) of the two QTL were 30.97% and 8.65%, respectively. Combined with gene annotation, mutation site analysis, and real-time quantitative polymerase chain reaction (qRT-PCR) analysis, one candidate gene in A08 was identified, namely *Bra020861*. And an insertion and deletion (InDel) marker (co-segregated) named Crr1-196 was developed based on the gene sequence. *Bra013275*, *Bra013299*, *Bra013336*, *Bra013339*, *Bra013341*, and *Bra013357* in A01 were the candidate genes that may confer clubroot resistance in Chinese cabbage. The resistance resource and the developed marker will be helpful in *Brassica* breeding programs.

## 1. Introduction

Chinese cabbage (*B*. ssp. *pekinensis*) is one of the most important vegetables in Asia and worldwide [1]. The obligate parasite *P. brassicae* is the causal agent of clubroot, a soil-borne disease that leads to severe global crop losses in Chinese cabbage and other *Brassicaceae* [2,3,4,5,6]. *P. brassicae* can be transmitted through various media, including seeds, soil, infected plant materials, water, and animal manure. The pathogen infects the host by wounds in the root hair and epidermis. The cortex is infected when the pathogen reaches the vascular system [7,8].

The susceptible host’s vascular system has severe distortion, with a significant damage to the xylem and an increase in meristematic activities within the vascular cambium (VC) and phloem parenchyma (PP) cells in the hypocotyl region. As a result, *P. brassicae* extensively colonizes and spreads to the underground parts of the infected plant through the vascular system, leading to the formation of club-shaped galls on the roots [7,9]. The above-ground parts of the infected plants will show wilting, stunting, leaf chlorosis, and growth retardation, which block the water and nutrition from root to shoot, ultimately causing plant death [10,11].

Clubroot is widely prevalent in Chinese cabbage and other brassicas worldwide [12]. Various chemical and biological control methods have been used to manage this destructive disease, but issues such as environmental pollution, poor soil quality, and ineffective results have emerged. Resistance breeding is considered the most effective strategy for managing this soilborne disease [5,13,14]. Identifying, mapping, and cloning resistance genes provide a strategy for quickly selecting and using new clubroot-resistant (CR) resources.

In *B. rapa* (A genome), several clubroot-resistant genes/loci have been reported in the recent years. They were located in chromosomes A01, A02, A03, A05, A06, and A08 in the A genome (*Brassica rapa* genome). Chromosome A03 was reported to be having the most CR loci/genes. These loci/genes are clustered in four location-specific regions: region I has 1.9–6.6 Mb, with *PbBa3.1* resistant to Pb2 in ‘ECD04’ [15]. Region II has 13.5–16.4 Mb including *PbBa3.2*, *Crr3*, *CRk*, and *CRd* [1,15,16,17]. Among them, *PbBa3.2* is resistant to Pb10 in ‘ECD04’ and *Crr3* is resistant to Ano-01 in ‘Milan White’. The CR locus *CRk* is derived from the fodder turnip ‘Debra’ and shows broad-spectrum resistance to most of the isolates. Region III has 18.4–19.6 Mb, with *PbBa3.3* resistant to Pb7 in ECD04. Region IV has 23.5–27.3 Mb including *CRa*, *CRb*, *Rcr1*, *Rcr4*, and *Rcr5* [18,19,20]. The dominant CR gene *CRa* derived from the fodder turnip line ECD02 [21] is resistant to pathotype 2 (Pb2), which was the first cloned CR gene. The strong dominant CR gene *CRb* derived from the European fodder turnip ‘Gelria R’ [22] is resistant to Pb2, 3, 4, and 8 [19]. The CR gene *Rcr1* is derived from the pak choi cultivar ‘Flower Nabana’, with efficacy against Pb3. *Rcr4* is resistant to Pb2, 3, 5, 6 and 8 *Rcr5* is resistant to Pb3. These loci were located in this region [23]. *PbBa1.1* is resistant to Pb2 and 7 in A01. The CR locus *CRc* derived from th fodder turnip ‘Debra’ is located in A02 [17]. *Rcr8* and *Rcr9* are resistant to a new physiological race 5X, located on chromosomes A02 and A08, respectively [23]. The CR gene *CrrA5* is mapped on the chromosome A05. *Crr1*, *Crr2*, and *Crr4* from the CR turnip ‘Siloga’ [24] are located on chromosomes A08, A01, and A06, which are the major effect gene, modification gene, and minor effect gene showing resistance to the isolates of pb1, 2, and 4. *Crr1* is composed of two genes *Crr1a* and *Crr1b*, of which *Crr1a* was cloned [25]. *PbBa8.1* is resistant to Pb4 in A08. Although several loci/genes that confer resistance have been identified, *P. brassicae* has physiological specialization and rapid changes. As a result, the host’s resistance genes typically provide resistance to only a limited number of pathotypes, and a single resistance gene can be easily broken. The loss of effectiveness in clubroot-resistant cultivars has been found in regions around the world [13,22,26]. For comprehensive resistance to the occurrence of clubroot, identification of novel resistance loci/genes are often necessary to polymerization breeding.

Next-generation sequencing (NGS)-based bulked segregant analysis (BSA) is a powerful technique used for gene identification and mapping genes. It also helps to establish the association between agronomic traits and molecular markers, making it highly valuable in crop breeding [22,27,28,29,30,31]. This tool has been applied to the resistance of rice blast [32,33]; bacterial wilt (BW) [34,35]; downy mildew [36,37]; phytophthora capsica [38,39]; clubroot [40,41]; and other plant diseases. To enhance the effectiveness of QTL mapping, different statistical methods have been proposed, including MutMap [42], G Value [43], and ED [44], among others, which aim to reduce noise and highlight the effects of QTL.

Marker-assisted selection (MAS) strategy relies on the use of molecular markers, which are linked to CR loci/genes in breeding. One such marker is Kompetitive Allele Specific PCR (KASP), which is a high-throughput marker based on SNP genotyping technologies. KASP is widely utilized for various purposes, including genetic mapping, development of trait-specific markers, characterization of germplasm (such as assessing genetic diversity, genetic relationship, and population structure), and quality control analysis (such as determining genetic identity, genetic purity, and parentage verification) [45,46,47]. To address the challenges posed by co-existing pathotypes and the rapid emergence of new *P. brassicae* pathotypes in the field, it is crucial to identify novel CR loci/genes and design molecular markers associated with resistance to these pathotypes.

In our previous study, the Chinese cabbage inbred line Bap246 was found to exhibit resistance to pb 4 from Beijing. In this study, we found no polymorphisms by the identified molecular markers linked to genes identified previously in Bap246. NGS-BSA and QTL mapping methods were used to identify the CR genes in “Bap246”. This research’s findings can serve as a foundation for the breeding of new Chinese cabbage cultivars with enhanced resistance. 

## 2. Materials and Methods

### 2.1. Population Construction and Pathogen Isolates

Bap246, a clubroot-resistant (CR) Chinese cabbage inbred line [7], was crossed with 11 clubroot-susceptible *B. rapa* materials (Table S1) to evaluate its resistance inheritance. Bac1344 and Bae090 were selected from the above susceptible materials to be crossed with Bap246 to construct F_1_. Subsequently, individual plants of F1 from two cross combinations were used to make a backcross with both parents to construct BC_1_P_1_ (F_1_ × Bap246) and BC_1_P_2_ (F_1_ × Bac1344/Bae090). F_2_ (Bap246 × Bac1344) population was also constructed and used to map the resistance loci.

The plant materials analyzed in this research were sourced from the Institute of Vegetables and Flowers, Chinese Academy of Agricultural Sciences (IVF, CAAS), Beijing, China, and were grown in a greenhouse environment.

The field isolate was found in a farm with a high incidence of severe clubroot disease in Beijing, China. The classification of this isolate was presented as pb4 by using the differential system given by Williams (1966) [48]. The infected galls were collected and stored in −20 °C until required.

### 2.2. Pathogen Inoculation and Disease Evaluation

Resting spores were prepared as 1 × 10^8^ spores/L as previously described [7]. Seedlings were inoculated by using the root dip method as described by Johnston (1968) [49] with some modifications. In brief, seeds of P_1_, P_2_, F_1_, BC_1P1_, BC_1P2_, and F_2_ populations were placed on a moistened filter paper in Petri dishes and allowed to germinate at room temperature until the rootlets reached a length of 1 cm (3–4 days before inoculation). Then the rootlets were soaked in *P. brassicae* resting spore suspension with 1 × 10^8^ spores/mL for about 10 min and transplanted into plugs (54 cm × 28 cm × 6 cm) with one seedling per cell, filled with sterilized potting mix. The plants were maintained in the greenhouse at a temperature of 23–25 °C, along with a 16 h photoperiod. The plants were watered daily and fertilization was carried out every two weeks. “Juxin” was used as the susceptible cultivar while “ECD04” was used as the resistance-control. 

Six weeks after inoculation, the plants were pulled up, and the roots were washed and rated for disease development. The disease scale consisted scores of 0, 1, 3, 5, 7, and 9 as described by Liu et al. [7]. Plants with disease scores of 0 or 1 were classified as “resistance” while disease scores of 3 and 5 were MR, and 7 and 9 were classified as susceptible.

The severity ratings for each individual were utilized in the calculation of a disease index (DI) using the following [50]:

$$DI=\frac{\left(0\times n0\right)+\left(1\times n1\right)+\left(3\times n2\right)+\left(5\times n3\right)+\left(7\times n4\right)+\left(9\times n5\right)}{\left(N\times 9\right)\times 100}$$

where n0, n1, n2, n3, n4, and n5 indicate plants number rated as 0, 1, 3, 5, 7, and 9, respectively. N is the number of all evaluated plants.

Disease incidence was calculated using the formula below:

$$Disease\;Incidence=\frac{n2+n3+n4+n5}{N}\times 100\%$$

Based on the DI values, the paretal lines and the individual lines of the developed population were classified as follows: immune (I, DI = 0); highly resistant (HR, 0 < DI ≤ 10); resistant (R, 10 < DI ≤ 30); susceptible (S, 30 < DI ≤ 50); and highly susceptible (HS, DI > 50).

### 2.3. Analysis of Markers Linked to Identified CR Genes/Loci

To compare with the previously identified clubroot disease loci/genes, we conducted PCR amplification of 29 markers associated with these loci/genes (CRa, CRc, CRk, CRd, Crr1, Crr2, Rcr1, and CrrA5) [17,19,21,24,51,52,53,54]. These markers had already been mapped to the *B. rapa* A-genome chromosomes. The parental lines were screened for polymorphisms using these markers (Table S2). The PCR cycling conditions were as described by Fredua-Agyeman et al. in 2017 [13]. The amplified products were then rubbed on agarose gel (2%) or 6% polyacrylamide gel to detect polymorphisms in the parental lines.

### 2.4. Bulk Segregant Analysis Sequencing (BSA-Seq)

Under greenhouse conditions, leaves of the three-week-old plants were collected from two parental lines (Bap246 and Bac1344), as well as the F_1_ and F_2_ population. These leaves were used for genome sequencing, with 347 F_2_ individuals utilized for BSA-seq and 849 F_2_ individuals used for mapping. Genomic DNA was extracted using the cetyl trimethylammonium bromide (CTAB) method, and its concentration was detected by using the NanoDrop 2000 spectrophotometer (Thermo Fisher Scientific, Waltham, MA, USA). Thirty individuals that exhibited immune/high-resistant (I/HR) or highly susceptible (HS) traits were selected to construct R/S pools. Equal amounts of genomic DNA from each pool were bulked and sequenced using the Illumina HiSeq 4000 system (Illumina, San Diego, CA, USA).

The genome sequencing of two parental lines, as well as R- and S-pools, and subsequent statistical analysis of the bioinformatics data followed the standard protocol provided by Illumina. A library with a 350 bp insert size was constructed and subjected to paired-end sequencing on an Illumina HiSeq platform. Raw data were filtered to remove bad reads and to ensure that quality data were used for further analysis. The resulting clean data were then subjected to statistical analysis to assess their quantity and quality, including parameters such as Q20, Q30, clean reads, base content statistics, and the CG content. To obtain the consensus sequence, the clean reads were aligned against the reference genome sequence “Chiifu-401-42” from the Ensemble Genome database using Burrows–Wheeler Aligner v.0.7.12 [55]. Reads from the R-pool and S-pool were separately aligned to the consensus sequence reads of Bap246 and Bac1344 for single nucleotide polymorphism (SNP) and insertion–deletion (InDel) calling using The Genome Analysis Toolkit (GATK) [56]. Heterozygote alleles in the parental were filtered out and retained for further analysis. Annotation of all qualified variants based on the GFF file was performed using the efficient tool, ANNOVAR, in perl language [57].

### 2.5. Association Mapping Analysis and Target Region Annotation

To conduct association mapping, two algorithms were utilized: the Euclidean distance (ED) [44] and the SNP/InDel index algorithm [43]. The ED algorithm was employed to calculate the allele frequency differences between the R- and S-pools for each SNP/InDel, following the methodology described by Hill et al. [44]. The calculation was performed using the equation provided below:

$$ED=\sqrt{\left\{{\left(A_{mut}-A_{wt}\right)}^{2}+{\left(Cmut-Cwt\right)}^{2}+{\left(Gmut-Gwt\right)}^{2}+{\left(Tmut-Twt\right)}^{2}\right\}}$$

To determine the frequency of DNA nucleotides (A, C, G, T), each letter was used as an indicator. To reduce noise and amplify the impact of large ED values, the ED values were squared. The data were then fitted using LOESS regression. The significance threshold for marker–trait associations was set at the median + 3SD of the LOESS-fitted values [44]. Regions of the genome where the LOESS-fitted values exceeded the threshold were identified as candidate regions associated with resistance to clubroot in *B. rapa*.

The ∆(SNP/InDel index) statistic, calculated as shown below, was used to find the significant differences in genotype frequency between the pools [43]. M represents Bac1344 (CS parent) and P represents Bap246 (CR parent). The genotype of the CS parent is denoted as “aa”, while the genotype of the CR parent is denoted as “ab”. The SNP/InDel index indicates the proportion of reads with SNPs/InDels that differ from the reference sequence. The ∆(SNP/InDel index) was calculated as follows:

$$SNP\;index\left(aa\right)=\frac{Maa}{Maa+Paa}$$

$$SNP\;index\left(ab\right)=\frac{Mab}{Mab+Pab}$$

$$∆\left(SNP/InDel\;index\right)=SNP/InDel\;index\left(aa\right)-SNP/InDel\;index\left(ab\right)$$

In this BSA-seq project, Maa stands for the depth of the aa population derived from Bac1344 (CS parent) and Paa stands for the depth of the aa population derived from Bap246 (CR parent). The Δ(SNP/InDel index) value is determined based on certain conditions: it is equal to 1 if the bulked DNA comprises only the parent Bac1344, −1 if it comprises only the parent Bap246, and 0 if the bulked DNA has the same SNP/InDel index as both parents in the genome region. To eliminate false positive sites, the Δ(SNP/InDel-index) values marked on the same chromosome can be fitted using the position of the marker on the genome. In this project, the DISTANCE method is used for fitting the Δ(SNP/InDel index), and then the area above the association threshold is selected as the region related to the trait.

To plot the average SNP/InDel index against the position of each sliding window (4 Mb) in the *B. rapa* 1.5 genome, the ∆(SNP/InDel index) data are then fitted using LOESS regression. The threshold for the significance of marker–trait associations was set at 1% of the largest Loess-fitted values [43]. Regions where the ∆(SNP/InDel index) values exceed the threshold are considered candidate regions (potential QTL) associated with resistance to clubroot. Sometimes, the threshold is lowered to identify a more likely location area and make full use of the data. Finally, ED, SNP/InDel index, and ∆(SNP/InDel index) values are plotted, and the intersections between the candidate regions identified using the ED and SNP/InDel index methods are designated as the final candidate regions associated with clubroot resistance.

### 2.6. SNP/InDel Markers Development and Genotyping in Target Region

Markers were developed by converting the polymorphic SNP/InDel identified within the target regions for clubroot resistance.

For each SNP, Primer3 software was used to design two allele-specific forward primers and one common reverse primer. The resulting KASP primers were then used to test the parents, the F_1_ and F_2_ populations. The genotyping data were analyzed using the SNPviewer software from LGC Genomics (http://www.lgcgenomics.com (accessed on 16 May 2023)) and viewed as cluster plot T.

For each InDel, the DNAMAN software was used to design one forward primer and one reverse primer. These InDel primers were used to test the parents, the F_1_ and F_2_ populations. The cycling conditions used were as follows: 3 min at 95 °C, followed by 35 cycles of 15 s at 95 °C, 55 °C, and 72 °C, with a final extension step at 72 °C for 5 min. After that, polypropylene gel electrophoresis was performed, and the bands were checked with a gel illuminator.

The significance of the correlation coefficients between the genotype and phenotype was determined using *t*-tests. Linkage groups were performed using QTL IciMapping 4.1 [58].

### 2.7. Expression Analysis

To assess the expression of the candidate genes in the root, stem, and leaf of Bap246 and Bac1344, real-time quantitative PCR (qRT-PCR) was performed. The total RNA was extracted from the samples using the RNApure Plant Kit (DNase I) (Vazyme, Nanjing, China). Following the manufacturer’s instructions, first-strand cDNA was synthesized using the SuperRT cDNA Synthesis Kit (Vazyme, Nanjing, China). The reference gene used was *Actin*, a housekeeping gene. qRT-PCR was performed in triplicate on the StepOnePlus Real-Time PCR System (Thermo Fisher, Waltham, MA, USA) using the ChamQ Universal SYBR qPCR Master Mix (Vazyme, Nanjing, China). Statistical analysis was carried out the well-known 2^−∆∆CT^ method [59]. The data are presented as mean *±* standard deviation.

## 3. Results

### 3.1. Phenotype Evaluation of P. brassicae Infection in Different Populations

The resistance of Bap246 was shown to be recessive in a cross with a susceptible pak choi Bac1344 [7]. To test the genetic pattern of Bap246 in different subspecies of *B. rapa*, Bap246 was crossed with eleven different germplasms of *B. rapa* lines, and F_1_ was inoculated with pb4 from Beijing, China by root soaking. All 11 F_1_ combinations were susceptible to *P. brassicae*, and the disease index (DI) was 54.22–100 respectively (Table 1). The resistance to clubroot in Bap246 was determined by recessive gene(s) by testing with different subspecies.

Based on the resistance of F_1_ and F_2_ results in previous research, more populations were constructed to test the resistance pattern (Table 2). The reciprocal cross of F_1_ showed both as susceptible (DI = 90.48 and 87.39, separately), which means the resistance was not due to maternal inheritance. The BC_1P1_ and BC_1P2_ populations were constructed with Bap246 and Bac1344. All plants of BC_1P2_ were susceptible (DI = 96.30). Among 52 BC_1P1_ plants, the ratio of resistance to susceptibility was 20:24 (scales 0 and 1 represent resistance and scales 3 to 9 represent susceptible).

Bae090 was also selected from the above susceptible materials to construct F_1_, F_1_′, BC_1P1_ (F1 × Bap246), BC_1P2_ (F_1_ × Bae090), and F_2_ populations with Bap246. Plants from the reciprocal cross of F_1_ were susceptible (DI = 87.41 and 91.50, separately). The resistance of BC_1P2_ plants were all susceptible (DI = 96.30), and BC_1P1_ showed the ratio of resistance to susceptibility of 29:22 separately. Among 664 F_2_ plants, the ratio of resistance to susceptibility was 155:509 (Table 3).

### 3.2. Identification of Markers Linked to CR Genes/Loci and Whole Genome Sequencing Analysis

Two parents in this study did not exhibit any polymorphism to markers linked to identified CR genes/loci. To identify the loci controlling CR in the F_2_ population (Bap246 × Bac1344), BSA-seq was employed. Genome-wide sequencing was conducted on two extreme pools (30 extremely resistant/susceptible individuals from F_2_ population with 347 individuals) with a 30-fold depth as well as 20-fold for the two parents. Approximately 51.64 Gb clean bases after filtering (Table 4) were generated with Illumina high-throughput sequencing, including 10.23 G clean bases for Bap246, 10.89 G bases for Bac1344, 15.38 G bases for the R-pool, and 15.14 G bases for the S-pool.

The average Q30 score of the reads was 93.25%, indicating that the sequence quality was high. On average, 96.95% of the clean reads from each library were successfully mapped to the *B. rapa* reference genome. The average depth of coverage was 21 fold in Bap246, 22 fold in Bac1344, 29 fold in the R-pool, and 30 fold in the S-pool, respectively. The high-quality data guaranteed the subsequent analysis (Table 4).

### 3.3. Association Analysis

**Figure 1:**
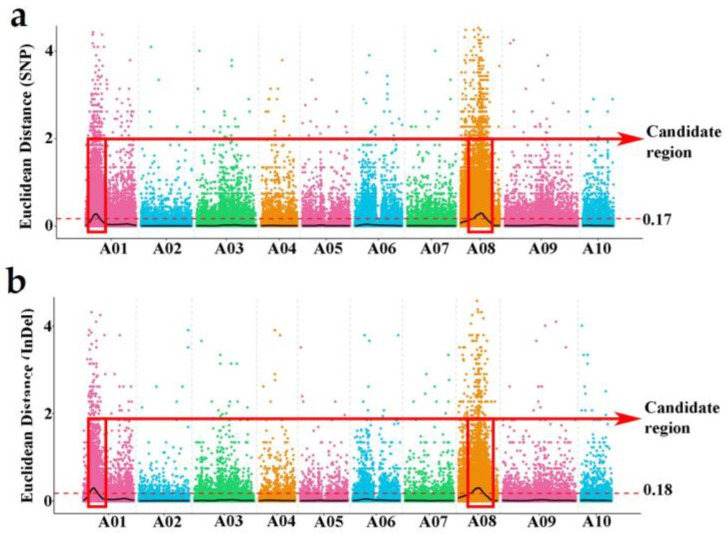
ED Manhattan plots graph of SNP (**a**) and InDel (**b**). The horizontal axis represents the chromosome number; the colored dots on the graph indicate the ED value of each SNP/InDel site; the black line represents the fitted ED value; the significance association threshold is depicted by a red dashed line. A higher ED value indicates a stronger association effect at that point. Two candidate regions associated with CR are identified on chromosomes A01 and A08, which are highlighted by red boxes. The marker–trait association thresholds are at 0.17 and 0.18, respectively.

Prior to analysis, the sequence data underwent trimming and filtering. After filtering, a total of 938,294 polymorphic SNPs and 329,281 polymorphic InDels were retained as useful markers. The purpose of this analysis was to identify candidate regions associated with clubroot resistance in Chinese cabbage using the ED method [44] and the SNP/InDel index [43] method. The Euclidean distance (ED) method used sequence data to identify target regions from R/S pools that are associated with specific traits. Non-target loci should have an ED value tending towards 0 while target loci exhibit higher values. A larger ED value indicates a greater difference between the two pools for a particular marker. For the ED method in SNP, when the marker–trait association threshold was 0.17, two candidate regions associated with clubroot resistance were identified on chromosomes A01 and A08 (Figure 1a). On the other hand, when applying the InDel-based ED method with a marker–trait association threshold of 0.18, one candidate region on chromosome A01 and four candidate regions on chromosome A08 were detected. These regions were found to be related to clubroot resistance (Figure 1b).

**Figure 2:**
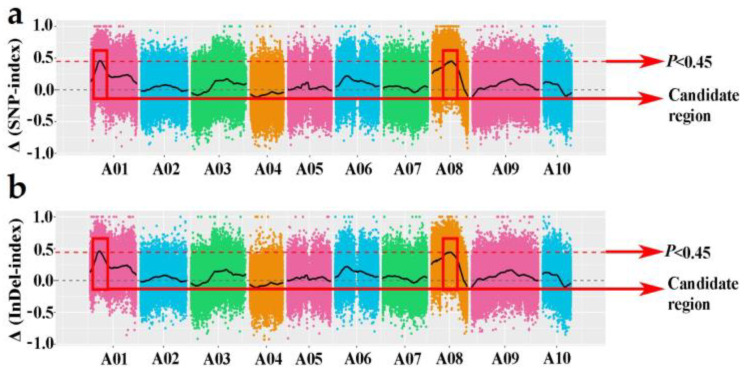
Genome wide ∆(SNP index) (**a**) and ∆(InDel index). (**b**) Manhattan plots graph of two extreme pools. The horizontal axis represents the chromosome, and the colored dots represent the calculated SNP/InDel ∆index; the black line is the average value of ∆(SNP/InDel index) in a sliding window of 1 Mb. The red dotted line represents statistical confidence intervals *p* < 0.45. Two regions on chromosomes A01 and A08 (the horizontal dotted line) are identified by red boxes.

By employing the SNP-index and InDel index methods for correlation analysis, a threshold value of 0.45 was set to determine the correlation between the R- and S-pools. Consequently, two potential regions on chromosome A01 and A08 (Figure 2a) were identified using the SNP-based ED method. Similarly, the InDel-index method yielded two candidate regions on the same part of the chromosomes A01 and A08 (Figure 2b), which coincided with the regions identified by the InDel-based ED method.

Two integration candidate regions of different methods were identified on chromosome A01 and A08, including the intervals 4,670,000–6,060,000 (A01) and 10,420,000–11,430,000 (A08), named *Cr4Ba1.1* and *Cr4Ba8.1*, respectively. These regions were considered to be verified as associated with CR.

A total of 329 genes were annotated within the candidate region, with 196 non-synonymous mutant genes annotated between parents and 44 frameshift mutant genes annotated. Subsequently, KEGG enrichment analysis was performed on the genes, and we found a total of 89 candidate genes annotated, divided into five categories: 10 genes were associated with cellular processes, 5 genes were associated with environmental information processing, 24 genes were associated with genetic information processing, 47 genes were associated with metabolism, and 3 genes are associated with organizational systems.

### 3.4. QTL Mapping of Cr4Ba8.1

**Figure 3:**
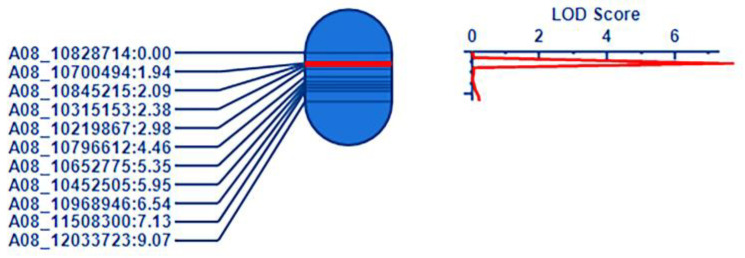
QTL mapping of *Cr4Ba8.1* on chromosome A08. Note: a partial linkage map on the left showed the candidate region of *Cr4Ba8.1* in red with markers on the **left**; the LOD score of QTL is shown on the **right**.

In *Cr4Ba8.1* (10.42–11.43 Mb), eleven SNPs were selected on the base of the large region containing the above region on chromosome A08, which were used to confirm the candidate region in the F_2_ population (Table S3). QTL IciMapping 4.1 was used for linkage analysis of the genotype. The result showed that 11 pairs of markers were distributed at the physical position on chromosome A08. The genetic distance and the phenotype of 849 individual plants in the F_2_ population were further integrated to narrow the candidate region. Finally, *Cr4Ba8.1* was located between the SNPs of A08-10700494 and A08-10845219, and the corresponding physical location was 10,700,494–10,845,219 bp in A08, which explained 8.65% of the phenotypic variation in clubroot (Figure 3).

### 3.5. Candidate Gene Prediction of Cr4Ba8.1

Combined with the transcriptome and genome data in BRAD, a total of 18 annotated genes in the target regions of *Cr4Ba8.1* were named *Bra020858-Bra020876* (Table S4). These genes were further analyzed for their potential involvement in fungal disease resistance. It encodes a Toll-Interleukin-receptor/nucleotide binding site/leucine-rich repeat (TIR-NBS-LRR class) disease resistance protein. The expression level of *Bra020861* was higher in the root compared to the leaf and stem in *Arabidopsis* (Table 5). *Bra020876*, which is homologous to *AT4G20940* in *Arabidopsis*, was annotated as having an LRR domain. Furthermore, *Bra020860*, homologous to *AT4G22060* in *Arabidopsis* and annotated as having an F-box domain, was considered a functional gene associated with CR. The sequence of *Bra020861* has multiple non-synonymous mutations and indel mutations (Table 6).

**Figure 4:**
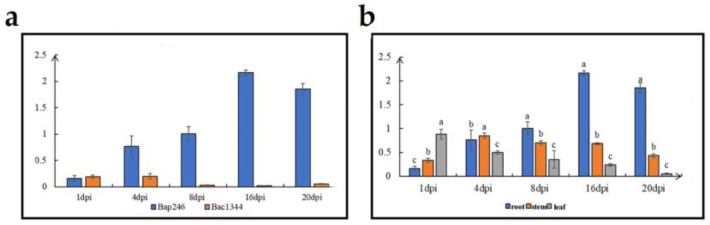
The q-PCR of *Bra020861* in parents. (**a**): gene expression of target gene in the roots of CR material Bap246 (blue bars)/CS material Bac1344 (orange bars) at 1, 4, 8 16, and 20 dpi; (**b**): gene expression of root (blue bars)/stem (orange bars)/leaf (grey bars) of clubroot-resistant material Bap246 at 1, 4, 8 16, and 20 dpi.

qRT-PCR analysis was conducted to detect *Bra020861* expression at 1, 4, 8, 16 and 20 d after inoculation with *P. brassicae* in root, stem, and leaf of Bap246, with Bac1344 as the control of the root. The results showed that *Bra020861* were up-regulated significantly after *P. brassicae* inoculated in Bap246 after 4 dpi and showed the highest expression at 16 dpi in Bap246 (Figure 4a). The expression level in the root in Bap246 was higher compared to the levels observed in the leaf and stem (Figure 4b). Based on these results, *Bra020861* may be the candidate gene of *Cr4Ba8.1*. *Bra020861* encodes the TIR-NBS-LRR domain for resistance, which participated in an important role in effector-triggered immunity (ETI).

### 3.6. Molecular Marker of Bra020861

**Figure 5:**
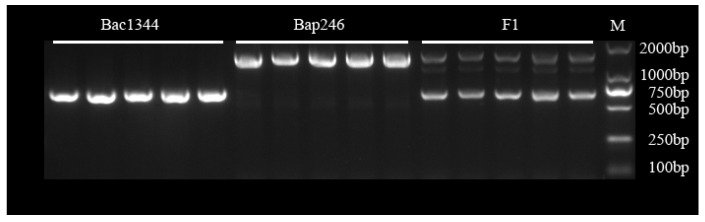
Agarose gel electrophoresis profiles of InDel markers (*Crr1*-196). Lanes 1–5 were individuals from CS parent Bac1344 showing a ~750 bp band; lanes 6–10 were individuals from CR parent Bap246 showing a ~1500 bp band; lanes 11–15 were individuals from F1 showing heterozygous band; marker is shown on the right with the size marked on the right.

Based on the SNPs/InDels of *Bra020861* and the sequence of *Crr1*, InDel marker on the exon1 of *Bra020861* was developed. This marker proved to be valuable in predicting the phenotypes of the offspring of Bap246 × Bac1344. Agarose gel electrophoresis showed that the amplified band size of the indel marker *Crr1*-196 was about 1650 bp in the resistant parent Bap246 and about 750 bp in the susceptible parent Bac1344, and heterozygous bands appear in F_1_ (Figure 5). Cloning of the PCR products showed that Bap246 had a 902 bp insertion on exon1, indicating that the molecular marker designed in this study was specific and could accurately identify *Bra020861*.

### 3.7. QTL Mapping of Cr4Ba1.1

**Figure 6:**
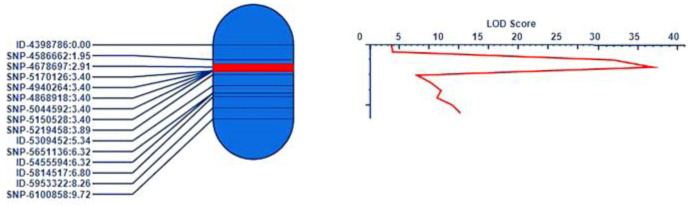
QTL mapping of *Cr4Ba1.1* on chromosome A01. A partial linkage map on the left shows the candidate region of *Cr4Ba1.1* in red with markers on the **left**; the LOD score of QTL is shown on the **right**.

In *Cr4Ba1.1* (4.67–6.06 Mb), ten SNPs and five InDels were designed based on the larger region containing the above region on chromosome A01, which were used to screen the recombinant individuals of the F_2_ population (Table S3). QTL IciMapping 4.1 was used for linkage analysis of the genotype. The result showed that 15 pairs of markers were distributed at the physical position on A01 chromosome. Finally, *Cr4Ba1.1* was located between the makers of SNP-4678697 and SNP-5170126, and the corresponding physical location was 4,678,697–5,170,126 bp in A01, which explained 30.97% of the phenotypic variation in clubroot (Figure 6).

### 3.8. Candidate Gene Prediction of Cr4Ba1.1

Similarity, combined with the transcriptome data and BRAD database, a comprehensive analysis revealed that a total of 85 annotated genes covered the target regions of *Cr4Ba1.1*, which were named *Bra013273*-*Bra013357*. Among these genes, twelve were identified to potentially play a role in disease resistance and were selected for further investigation. This subset included four R genes (*Bra013336*, *Bra013339*, *Bra013345*, and *Bra013357*) and eight functional genes associated with CR (*Bra013275*, *Bra013281*, *Bra013289*, *Bra013299*, *Bra013310*, *Bra013341*, *Bra013347*, and *Bra013348*) (Table S5). By sequencing data and bioinformatics analysis, combined with gene annotation and expression parts, *Bra013275*, *Bra013281*, *Bra013289*, *Bra013299*, *Bra013336*, *Bra013339*, *Bra013341, Br013345*, and *Bra013357* have multiple non-synonymous mutations and indel mutations, which are the candidate genes for further analysis (Table 7).

### 3.9. Expression Characteristic Analysis of Cr4Ba1.1 Candidate Genes

**Figure 7:**
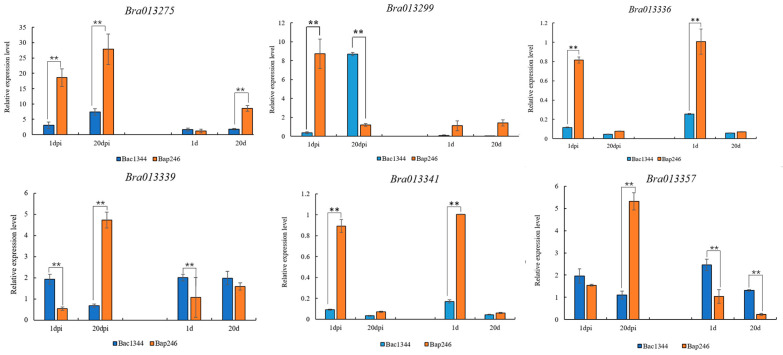
The q-PCR of candidate genes in parents of *Cr4Ba1.1*. Note: gene expression in the roots of CR material Bap246 (orange bars)/CS material Bac1344 (blue bars) at 1 and 20 dpi; gene expression with inoculated materials on the left and with non-inoculated materials on the right; ** indicate genes expression have extremely significant different between two parents.

To verify the candidate genes for Bap246 in A01, qRT-PCR analysis was conducted to assess the expression of these candidate genes at 1- and 20-days post-inoculation (dpi) with *P. brassicae* in the roots of both Bap246 and Bac1344, with non-inoculated plants serving as the control (Table S6). The results showed that three genes (*Bra013299*, *Bra013336*, and *Bra013341*) were up-regulated significantly at 1 dpi in Bap246; two genes (*Bra013339* and *Bra013357*) were up-regulated significantly at 20 dpi in Bap246; one gene (*Bra013275*) was up-regulated significantly at 1 and 20 dpi in Bap246 (Figure 7). Among them, *Bra013299* is homologous to *AT4G18360* in Arabidopsis, which is annotated as the defense response signaling pathway. *Bra013336* is homologous to *AT4G18640* in Arabidopsis and is associated with the leucine-rich repeat N-terminal domain. *Bra013341* is homologous to *AT4G18710* in *Arabidopsis*, which plays a role in regulating auxin and brassinolide signaling pathways, as well as influencing the expression of root epidermal cells. *Bra013339* corresponds to *AT4G18670* in *Arabidopsis* and is annotated as having the leucine-rich repeat N-terminal domain. *Bra013357* is homologous to *AT4G19920* in *Arabidopsis* and is annotated as a disease-resistant protein. Therefore, *Bra013275*, *Bra013299*, *Bra013336*, *Bra013339*, *Bra013341*, and *Bra013357* were considered as functional genes associated with clubroot resistance in A01.

## 4. Discussion

### 4.1. Rapid Mapping Strategy of CR Loci

Rapid and effective mapping of disease-resistance genes is not only the starting point of basic research but also is the important foundation of applied research. Map-based cloning with genome random markers is a time-consuming method and requires genetic map and markers associated with physical positions to identified the target gene on the areas of chromosomes. The advancement of sequencing technology has played a significant role in the success and precision of gene identification, with BSA-Seq being a key contributor to this progress.

In this study, the Chinese cabbage line Bap246, with resistance to pb 4 (the most common physiological pathotype in China) from Beijing, China, was used as the resistant parent, and the F_2_ population (Bap246 × Bac1344) was used for mapping by the BSA-Seq method. To emphasize the importance of the positioning interval for improved noise reduction, two suitable statistical methods were employed to identify two significant peaks at *Cr4Ba1.1* and *Cr4Ba8.1*. ED increase in CR sensitivity near the high allele frequency locus in the R-pool suggests enhanced effectiveness. Additionally, G’ is anticipated to decline more rapidly around the causal site, indicating narrower intervals of support surrounding QTL [43].

The utilization of KASP analysis in QTL mapping is an effective method for accurately pinpointing disease-resistant genetic loci. KASP is one of the SNP typing markers that has been widely used. The utilization of KASP markers offers several benefits, including high throughput, cost-effectiveness, flexibility, and speed. In our research, we identified a total of 938,294 SNPs and 329,281 InDels with polymorphisms between the parents. Additionally, KASP markers linked to CR were obtained and constructed in the QTL map. Through BSA-Seq analysis, numerous SNP/InDel markers were developed within the candidate region for *Cr4Ba1.1* and *Cr4Ba8.1* sites. Then, the software QTL Icimapping 4.1 and large populations were used with KASP and InDel markers to further narrow the candidate region. *Cr4Ba1.1* and *Cr4Ba8.1* explained 30.97% and 8.65% of the phenotypic variation in clubroot. These two loci variations were not high, indicating that they may have minor genes. Fine mapping in populations having more than 10,000 individuals in the next step will narrow the candidate region, which may enhance the variations in these two loci. Our study utilizes an efficient method for molecular marker-assisted selection in the identification of CR in *B. rapa*.

### 4.2. The CR Mechanism of Bap246

CR gene *Bra020861* showed polymorphisms between the two parents, which is homologous to the identified CR locus *Crr1a* in *B. rapa*. In our research, we screened existing CR genes in Bap246 using identified molecular markers and did not find polymorphism in Bap246. Subsequently, we designed molecular markers (*Crr1*-196-InDel) in *Bra020861* based on the sequencing information and verified the presence of CR gene *Bra020861* in Bap246.

In a previous study, *Crr1* locus was subjected to fine mapping, which led to the discovery of two genes named *Crr1a* and *Crr1b*. *Crr1a* is identified as the major gene responsible for clubroot resistance [25]. *Crr1a* encodes a disease-resistant protein of the TIR-NBS-LRR type and is expressed in the stele or cortex of hypocotyl and roots, specifically in the region at the secondary infection phase [25]. Jahn et al. (2013) [60] found that the regulation of auxin- and cytokinin-related genes was initiated at 20 days after inoculation in Arabidopsis (secondary infection stage). The expression analysis of *Cr4Ba8.1* showed that high expression in the resistant parents reached 16 days after inoculation, which mainly regulated the related defense mechanisms in secondary infection. However, the genetic analysis of an F_2_ population resulting from a cross between G004 (CS DH line) and A9709 (CR DH line) revealed that Crr1 exhibits incomplete dominance. It was observed that having a heterozygous *Crr1* locus alone was not enough to confer complete resistance to pathotype 4. The hypothesis is that the presence of a small amount of susceptible *Crr1a* protein functions as a dominant-negative regulator in heterozygous plants [25]. In our study, F_1_ was highly susceptible after inoculation. One of the reasons may be that *Crr1* is not completely dominant or has low expression, resulting in F_1_ being susceptible. On the other hand, we found two QTL loci in BSA-Seq. *Cr4Ba1.1* located on chromosome A01 may have an epistatic effect and mask the functional expression of *Crr1*.

### 4.3. The Interaction between Cr4Ba1.1 and Cr4Ba8.1

In our study, the candidate gene of *Cr4Ba8.1* was homologous to *Crr1a*. Previous studies showed that *Crr1* (A08) and *Crr2* (A01) are complementary for clubroot dominant resistance. The resistance to clubroot was significantly higher when both loci had homozygous resistant alleles, compared to when they had heterozygous alleles [24].

The candidate gene on *Cr4Ba1.1* is not *Crr2*. By using the linkage marker (BRMS-096) of *Crr2*, we found that there was no polymorphism between the CR parent Bap246 and the CS parent Bac1344. Then, the Bap246 test was crossed with several susceptible materials and F_1_ was shown to be susceptible. Finally, *Cr4Ba1.1* was different from *Crr2*. These results confirm that we have identified a novel CR locus.

To minimize economic losses, extensive research has been conducted on several dominant clubroot (CR) genes/loci in Chinese cabbage, *CRa*, *Crr1*, and *CRb* [51,52,61]. The majority of these disease-resistant genes are dominant and code for NBS-LRR proteins, which provide race-specific resistance against pathogens [62,63]. In the resistance system of clubroot, the regulation of the R gene encodes NBS-LRR resistant protein, which plays an extremely important role in controlling the activity of plant disease-resistance proteins [51,61,64]. In this study, the two CR loci interaction causes the recessive resistance interaction, which was not found, indicating a new mechanism of clubroot resistance in these materials may exist. Verification of the candidate genes and transcriptome analysis will be needed for the next analyses.

For the present analysis of the mechanism of clubroot resistance, transcription factors (TFs), as molecular switches that control the expression of stress-responsive genes, play a crucial role in regulating various abiotic stress responses. Various families of transcription factors, including MYB, bHLH, WRKY, NAC, etc., have been identified in different plant crops. The WRKY family of transcription factors has been found in the promoters of numerous plant defense-related genes that are involved in responding to biotic and abiotic stresses [65]. Current evidence suggests that many WRKY proteins play a regulatory role in the response to pathogen infection and other stressors [66]. Recent studies have indicated that F-box proteins may up-regulate the expression of defense-related genes in rice [67] and contribute to cell death and defense responses triggered during pathogen recognition in tobacco and tomato [68]. Plant hormones also play a crucial role in disease resistance. Auxin signaling is important in the interaction between many plants and pathogens, as it regulates various processes related to pathogen growth [69,70]. After rhizobia infection, the cell division process involves simultaneous activities of auxin, cytokinin, and brassinosteroids [60]. Salicylic acid (SA), jasmonic acid (Ja), and ethylene (ET) are the primary regulators that induce the defense signal network in plants, regulating the defense signals of plant roots and even the whole plant [71].

To determine the candidate genes, further narrowing down of the genomic intervals of QTL is still required. The identification of candidate genes from this study offers valuable insights into the genetic mechanism underlying clubroot resistance in *B. rapa*.

## 5. Conclusions

In this study, Bap246 was tested and showed recessive resistance to clubroot. An F_2_ population was derived to locate the QTL. Two QTL on chromosomes A01 (4.67–6.06 Mb) and A08 (10.42–11.43 Mb) were found and named *Cr4Ba1.1* and *Cr4Ba8.1*, respectively. Using KASP and InDel markers, we narrowed the target regions to 4.67–5.17 Mb (A01) and 10.70–10.84 Mb (A08). Two QTL phenotypic variations explained (PVE) were 30.97% and 8.65%, respectively. One candidate gene in A08 was identified, namely *Bra020861*. Six candidate genes in A01 were detected, which may confer clubroot resistance in Chinese cabbage.

## Figures and Tables

**Table 1:** Disease evaluations of CR in Bap246 × eleven Chinese cabbage germplasms.

Materials		F_1_								
P_1_	P_2_	Total	Disease Grades						Disease Index (DI)	Resistance Level
			0	1	3	5	7	9		
Bap246	Taqing	50	0	0	0	3	5	42	95.11	HS
	Quanmei	50	0	0	0	0	10	40	95.56	HS
	Heatwave	53	0	0	15	23	13	2	56.39	S
	Bak10	48	2	0	0	0	4	42	93.98	HS
	Bak11	50	0	0	0	0	4	46	98.22	HS
	Bak12	50	0	0	0	0	0	50	100.00	HS
	Bac1343	50	0	0	22	11	15	2	54.22	S
	Bac1344	30	0	0	2	0	13	15	90.48	HS
	Bae090	43	0	0	0	0	11	32	85.93	HS
	Bae091	50	0	0	0	1	20	29	90.22	HS
	Bae092	52	0	0	0	2	4	46	96.58	HS

**Table 2:** Disease evaluations of CR in Bap246 × Bac1344.

Generation	Total	Disease Grades						DI
		0	1	3	5	7	9	
Bap246 (P_1_)	48	39	9	0	0	0	0	0.02
Bac1344 (P_2_)	33	0	0	0	3	7	23	91.25
F_1_	35	0	0	0	5	5	25	90.48
F_1_′	37	0	0	0	7	7	23	87.39
BC_1P1_	52	14	15	1	3	7	11	38.68
BC_1P2_	36	0	0	0	2	2	32	96.30
F_2_	849	130	76	85	136	203	219	57.62

**Table 3:** Disease evaluations of CR in Bap246 × Bae090.

Generation	Total	Disease Grades						DI
		0	1	3	5	7	9	
Bap246	48	39	9	0	0	0	0	0.02
Bae090	35	0	0	0	4	7	24	90.48
F_1_	30	0	0	0	2	13	15	87.41
F_1_′	34	0	0	0	0	13	21	91.50
BC_1_P_1_	44	15	5	0	2	12	10	47.73
BC_1_P_2_	36	0	0	0	2	2	32	96.30
F_2_	664	87	68	66	85	135	223	60.96

**Table 4:** Qualitative and quantitative statistics of sequencing data.

Sample Name	Clean Reads	Clean Bases (G)	CleanQ30 (%)	Clean GC Content (%)	Map Reads Rate (%)	Coverage Rate (%)	Average Depth
Bap246	34,184,318	10.23	93.21	36.59	97.35	93.53	21
Bac1344	36,363,047	10.89	93.29	37.47	96.44	91.29	22
R-pool	51,366,194	15.38	93.66	37.29	96.87	96.03	29
S-pool	50,591,219	15.14	92.86	37.53	97.14	95.95	30

**Table 5:** Candidate gene information of *Cr4Ba8.1*.

Gene ID	Homologous Gene	Annotation	*A. thaliana* Highest Expression
*Bra020860*	*AT4G22060*	F-box domain	Silique
*Bra020861*	*AT3G25510*	TIR domain; leucine-rich repeats (2/6 copies); NB-ARC domain	Root
*Bra020876*	*AT4G20940*	Leucine-rich repeats (2 copies)	Flower

**Table 6:** SNP/InDel of *Cr4Ba8.1* associated with clubroot resistance.

Gene ID	SNP		InDel			
	Synonymous Coding	Non-Synonymous Coding	Frameshift Mutation	Codon Deletion	Codon Insertion	Codon Change + Deletion
*Bra020860*	2	5	—	—	—	—
*Bra020861*	22	49	14	2	2	1
*Bra020876*	40	12	—	—	—	—

**Table 7:** SNP/InDel of *Cr4Ba1.1* associated with clubroot resistance.

Gene ID	SNP		InDel			
	Synonymous Coding	Non-Synonymous Coding	Frameshift Mutation	Codon Deletion	Codon Change + Deletion	Codon Change + Insertion
*Bra013275*	3	4	—	—	—	—
*Bra013281*	5	12	3	—	—	—
*Bra013289*	1	3	—	1	—	1
*Bra013299*	12	6	—	—	—	—
*Bra013310*	—	1	—	—	—	—
*Bra013336*	7	3	—	—	—	—
*Bra013339*	6	18	—	3	—	—
*Bra013341*	10	1	1	—	—	—
*Bra013345*	6	1	—	—	—	—
*Bra013347*	—	—	—	—	—	—
*Bra013348*	—	—	—	—	—	—
*Bra013357*	5	6	1	—	1	—

## Data Availability

Sequence data can be found in the NCBI databases under the following accession numbers: SRR27393538 (Bap246), SRR27393537 (Bac1344), SRR27393536 (R-pool), and SRR27393535 (S-pool).

## Supplementary Materials

The following supporting information can be downloaded at: https://www.mdpi.com/article/10.3390/genes15030274/s1. Table S1: Disease evaluations of clubroot resistance in Chinese cabbage materials; Table S2: Molecular markers linked to identified CR genes; Table S3: SNP/InDel marker primer information for localized genes; Table S4: Candidate gene information of *Cr4Ba8.1*; Table S5: Candidate gene information of *Cr4Ba1.1*; Table S6: The special primer sequences of candidate genes in A01 used in qRT-PCR.
