# Supplementary material for: Identification of Clubroot (Plasmodiophora brassicae) Resistance Loci in Chinese Cabbage (Brassica rapa ssp. pekinensis) with Recessive Character
Source: Genes (Basel). 2024 Feb 22;15(3):274. doi: 10.3390/genes15030274 (PMC10970103; doi:10.3390/genes15030274)
Supplement: Supplementary file 1 [file genes-15-00274-s001.zip › Table S1.pdf]

Table S1. Disease evaluations of clubroot resistance in Chinese cabbage materials

Number	Name	Type	Disease Index
1	Taqing	Chinese cabbage	90.58
2	Quanmei	Chinese cabbage	77.78
3	Heatwave	Chinese cabbage	100.00
4	Bak10	Chinese cabbage	90.58
5	Bak11	Chinese cabbage	97.62
6	Bak12	Chinese cabbage	100.00
7	Bac1343	pac choi	96.97
8	Bac1344	pac choi	100.00
9	Bae090	pac choi	100.00
10	Bae091	pac choi	92.03
11	Bae092	pac choi	95.56
