# Supplementary material for: Identification of Clubroot (Plasmodiophora brassicae) Resistance Loci in Chinese Cabbage (Brassica rapa ssp. pekinensis) with Recessive Character
Source: Genes (Basel). 2024 Feb 22;15(3):274. doi: 10.3390/genes15030274 (PMC10970103; doi:10.3390/genes15030274)
Supplement: Supplementary file 1 [file genes-15-00274-s001.zip › Table S2.pdf]

**Table S2.** Molecular markers linked identified CR genes

| Primer IDs | Gene/Loci | Sequence of primer |
|---|---|---|
| Craim-T | | F: TATATTAATGATAAAGCAGAAGAAGAAA |
| | | R: AATGCGACTGAGAAAGTTGTAG |
| GC1680 | | F: ATTCTACTTCCTTTTAGCTGCTCTAACCGC |
| | | R: TTCGTAATAATTGATTTGCATATCAATTGG |
| GC2360-1 | | F: CAGCACCAGCATAACCAGCTACAGTC |
| | *CRa* | R: AGAACTTTGCAAGTGGCTCAGATAAT |
| SC2930-T | | F: TAGACCTTTTTTTTGTCTTTTTTTTTAC |
| | | R: CTAAGGCCATAGAAATCAGGTC |
| SC2930-Q | | F: CAGACTAGACTTTTTGTCATTTAGACT |
| | | R: AAGGCCATAGAAATCAGGTC |
| HC352b-SCAR | | F: CTTTATAATGGCTACTATTTA |
| | | R: TGCTCATGAGTGTATAACTA |
| TCR79 | | F: TGACGTTCAATCAAAGCCTGA |
| | | R: TTTAGCAATCAAATGCAAATTCAA |
| TCR108 | | F: CGGATATTCGATCTGTGTTCA |
| | | R: AAAATGTATGTGTTTATGTGTTTCTGG |
| K-3 | *CRb* | F: CTTTGGATTGTTGACCTT |
| | | R: ATGTTGATGCTACTGAGAC |
| TCR09 | | F: GCAGCAACCGATAATATAAGGA |
| | | R: AACCAGAAGAAGAAAAACAAAAA |
| TCR05 | | F: AGAATCATGACCGGGGAAAT |
| | | R: GCAGCTAAGTCATCGACCAA |
| KBrH129J18R | *CRb*$^{Kato}$ | F: AGAGCAGAGTGAAACCAGAACT |
| | | R: GTTTCAGTTCAGTCAGGTTTTTGCAG |
| m6r | | F: CCTCTTGGAAAACCCATGAA |
| | | R: GCAATTATTGGCCTGTTCGT |
| B50-C9 | *CRc* | F: GATTCAATGCATTTCTCTCGAT |
| | | R: CGTATTATATCTCTTTCTCCATCCC |
| B50-6R | | F: AATGCATTTTCGCTCAACC |
| | | R: CGTATTATATCTCTTTCTCCATCCC |
| HC688-4-6 | | F: TCTCTGTATTGCGTTGACTG |
| | *CRk* | R: ATATGTTGAAGCCTATGTCT |
| HC688-4-7 | | F: TCTCTGTATTGCGTTGACTG |

| | | |
|---|---|---|
| | | R: AAATATATGTGAAGTCTTATGATC |
| yau376 | *CRd* | F: TGTCACCAGCGCATTATAGC |
| | | R: AAGGGAGGGAAGATGGGTTG |
| yau389 | | F: TCAAGTGGAGAGGGTCAGTC |
| | | R: CAGTCACGAGGTCCCTAACA |
| BRMS-088 | *Crr1* | F: TATCGGTACTGATTCGCTCTTCAAC |
| | | R: ATCGGTTGTTATTTGAGAGCAGATT |
| BRMS-096 | *Crr2* | F: AGTCGAGATCTCGTTCGTGTCTCCC |
| | | R: TGAAGAAGGATTGAAGCTGTTGTTG |
| OPC11-2S | *Crr3* | F: GTAACTTGGTACAGAACAGCATAG |
| | | R: ACTTGTCTAATGAATGATGATGG |
| OPC11-1S | | F: TTACAGCTGGACCAAGAACATAG |
| | | R: ATCGATGTTTGTGAGTCTCTACT |
| BrSTS-54 | | F: CGTATAGACATAGAAGACATGGAAGC |
| | | R: GTGTTTATGCTGATTCCTTCACAG |
| BrSTS-61 | | F: CCATCCGCAGGAGTTAAAGTTGTA |
| | | R: AGAAAACGGCTGAGGGTTACTAC |
| sN8591 | *Rcr1* | R: TTGTGGGCAGGAACAATACA |
| | | F: CTGGACGAGCCAAGCTAATC |
| Sr6340I | | R: ATAGTTGGGAATGTGGCTGC |
| | | F: CGGACACGAAATCAAACCTT |
