# Supplementary material for: Identification of Clubroot (Plasmodiophora brassicae) Resistance Loci in Chinese Cabbage (Brassica rapa ssp. pekinensis) with Recessive Character
Source: Genes (Basel). 2024 Feb 22;15(3):274. doi: 10.3390/genes15030274 (PMC10970103; doi:10.3390/genes15030274)
Supplement: Supplementary file 1 [file genes-15-00274-s001.zip › Table S3.pdf]

Table S3. SNP/InDel makers primers information for localized genes

Primer	Primer sequence
A08_10315153	Fa: GAAGGTGACCAAGTTCATGCTGAGCTAAATCCGAAAGAGAGAATCG<br>Fb: GAAGGTCGGAGTCAACGGATTAGAGCTAAATCCGAAAGAGAGAATCT<br>R: AAGAACGGTTACTGTTTTGATCCGAAAC
A08_10219867	Fa: GAAGGTGACCAAGTTCATGCTGGAGCATTTGTCTTATAACTGCATAC<br>Fb: GAAGGTCGGAGTCAACGGATTAGGAGCATTTGTCTTATAACTGCATAT<br>R: TACATAACCCTCCAAATAACATTTTCCATTG
A08_10452505	Fa: GAAGGTGACCAAGTTCATGCTTACTGCTCCACCGTATGAAAACAAA<br>Fb: GAAGGTCGGAGTCAACGGATTCTGCTCCACCGTATGAAAACAAG<br>R: CAGATAAGTCAAAACTCCCATCTGAATTG
A08_10700494	Fa: GAAGGTGACCAAGTTCATGCTTGATGTCTCCCCTCAAATCCAAC<br>Fb: GAAGGTCGGAGTCAACGGATTTGATGTCTCCCCTCAAATCCAAG<br>R: TTACTGTTATCGCCGTGGACTTTGC
A08_10796612	Fa: GAAGGTGACCAAGTTCATGCTCAATTAAGGTATACACGTTATTTATATTCAAC<br>Fb: GAAGGTCGGAGTCAACGGATTATCAATTAAGGTATACACGTTATTTATATTCAAT<br>R: TACGGGAAGTAAAATATTGAATTTTGACCCA
A08_10845215	Fa: GAAGGTGACCAAGTTCATGCTATCACTATAACTAATTAATATTTGAACTAAATTATAT<br>Fb: GAAGGTCGGAGTCAACGGATTTCACTATAACTAATTAATATTTGAACTAAATTATAC<br>R: CATTCGGATCCACATCAGAAATGTTTAC
A08_10968946	Fa: GAAGGTGACCAAGTTCATGCTAATAAAACTACGTTAGAAAAATTGCAACTTG<br>Fb: GAAGGTCGGAGTCAACGGATTATTAATAAAACTACGTTAGAAAAATTGCAACTTA<br>R: AACCACGTATAGTTTGCATGCATATTCC
A08_	Fa:

11508300	GAAGGTGACCAAGTTCATGCTGTCATCATTATATTGTAGTTAAGTCATTCTTT<br>Fb: GAAGGTCGGAGTCAACGGATTGTCATCATTATATTGTAGTTAAGTCATTCTTA<br>R: TGAACCTATGACCTCTTAGATCAAATTTAAC
A08_ 12033723	Fa: GAAGGTGACCAAGTTCATGCTAGATCTTTGGTAAAGATTTCATCGGG<br>Fb: GAAGGTCGGAGTCAACGGATTTAAGATCTTTGGTAAAGATTTCATCGGT<br>R: GGAATCATCAGCAGCTTCAGGATTAG
A08_ 10828714	Fa: GAAGGTGACCAAGTTCATGCTTTTGGGGGACGAGGCAATACTTT<br>Fb: GAAGGTCGGAGTCAACGGATTTGGGGGACGAGGCAATACTTC<br>R: ATTCCTTGGACAGCGTTTGCAAGCA
A08_ 10652775	Fa: GAAGGTGACCAAGTTCATGCTTTTATCAATCTCTGTGATTTGAGATCC<br>Fb: GAAGGTCGGAGTCAACGGATTTTTATCAATCTCTGTGATTTGAGATCG<br>R: CTAAACCCTAAAGTTTCCAACTTTTAGAATATC
SNP-4586662	Fa: GAAGGTGACCAAGTTCATGCTCTCGAGGATCTGAGATGAGATCT<br>Fb: GAAGGTCGGAGTCAACGGATTTCGAGGATCTGAGATGAGATCC<br>R: GGGTGCACTAGCTGATATATTCCTAAG
SNP-4678697	Fa: GAAGGTGACCAAGTTCATGCTTTTCTCCCGGTGAGAATATCTCC<br>Fb: GAAGGTCGGAGTCAACGGATTAATTTTCTCCCGGTGAGAATATCTCT<br>R: ATTTCAACGAAAACAAAAACGTGTCAGAATG
SNP-5170126	Fa: GAAGGTGACCAAGTTCATGCTCTTAGAATGAAGAGAGAGCCTAAGTA<br>Fb: GAAGGTCGGAGTCAACGGATTTTAGAATGAAGAGAGAGCCTAAGTC<br>R: CAGCTTTATCAATCAAAAACCAAACTTCATTG
SNP-4940264	Fa: GAAGGTGACCAAGTTCATGCTTGTTCTTTTGTTCATGAAAGTCAAACCA<br>Fb: GAAGGTCGGAGTCAACGGATTTGTTCTTTTGTTCATGAAAGTCAAACCT<br>R: GGACTAGTCTGTTTGGTTTCTACTCG
SNP-4868918	Fa: GAAGGTGACCAAGTTCATGCTGATTCTTCCTTGGCAGTTTCCAC<br>Fb: GAAGGTCGGAGTCAACGGATTGAGATTCTTCCTTGGCAGTTTCCAT<br>R: GTCTTGGAGAATGATGTTTCCTTGTTTG
SNP-5044592	Fa: GAAGGTGACCAAGTTCATGCTTTCTGGGAAGCCGTTGTTAGGT<br>Fb: GAAGGTCGGAGTCAACGGATTTCTGGGAAGCCGTTGTTAGGC<br>R: GAATCACCTTCTCCCGGAAACAAAG
SNP-5150528	Fa: GAAGGTGACCAAGTTCATGCTCAGTATCATTTCACTACAGGTTTGG<br>Fb: GAAGGTCGGAGTCAACGGATTCCAGTATCATTTCACTACAGGTTTGT

	R: CAAAGATTAACTAACTTATGCTAACAGAAAGAC
SNP-5219458	Fa: GAAGGTGACCAAGTTCATGCTCATGACCAGATTTACTTGCACCATG<br>Fb: GAAGGTCGGAGTCAACGGATTCATGACCAGATTTACTTGCACCATA<br>R: ACTACAGAGGAAAAGCTTGCAAGGG
SNP-5651136	Fa: GAAGGTGACCAAGTTCATGCTCAATGAAGACATACTTGATGAGAGGA<br>Fb: GAAGGTCGGAGTCAACGGATTCAATGAAGACATACTTGATGAGAGGT<br>R: GCTCTTTCTCTGATCATTTACTCTCTTTTG
SNP-6100858	Fa: GAAGGTGACCAAGTTCATGCTTTGCGGATTCAAGGTTTCCGCAT<br>Fb: GAAGGTCGGAGTCAACGGATTGCGGATTCAAGGTTTCCGCAC<br>R: GCGAGAGTACGTTGATAGGTATGTATG
ID-5309452	F: GAGCAGGAGTTCTTCAAGC<br>R: CTGGATTCTCATTGGTAAGAGT
ID-5455594	F: CAATGAGACCCAGTCGGA<br>R: TAGCTGCCAAAGAATATCACAG
ID-5814517	F: CTCTGTGATTGGGAACCG<br>R: GCTTTTGCAGGCTTGTATG
ID-5953322	F: GGAGGGTTCGTCATGTATATG<br>R: ATCGCTATGGGTACAAAACTAC
ID-4398786	F: CGACCTTCTGGTGCATGT<br>R: CATGGACTCGCAACCTATG
