# Supplementary material for: Identification of Clubroot (Plasmodiophora brassicae) Resistance Loci in Chinese Cabbage (Brassica rapa ssp. pekinensis) with Recessive Character
Source: Genes (Basel). 2024 Feb 22;15(3):274. doi: 10.3390/genes15030274 (PMC10970103; doi:10.3390/genes15030274)
Supplement: Supplementary file 1 [file genes-15-00274-s001.zip › Table S4.pdf]

Table S4. Candidate genes information of *Cr4Ba8.1*

Gene	Homologous gene	Functional annotation	Highest expression
Bra020858	*AT4G22100*	Carbohydrate transport and metabolism	—
Bra020859	*AT4G22080*	Pectate lyase; Right handed beta helix region	Root
Bra020860	*AT4G22060*	F-box domain	Silique
Bra020861	*AT3G25510*	TIR domain; Leucine Rich repeats (2/6 copies); NB-ARC domain	Root
Bra020862	*AT4G21910*	MatE	Flower
Bra020863	*AT4G21910*	MatE	Flower
Bra020864	*AT4G21895*	AT hook motif; DNA binding protein	Flower
Bra020865	*AT4G21870*	Hsp20/alpha crystallin family; Posttranslational modification, protein turnover, chaperones	Flower
Bra020866	*AT4G21865*	Unknown	Flower
Bra020867	—	Unknown	—
Bra020868	*AT4G21850*	SelR domain; Peptide methionine sulfoxide red uctase B9	Root
Bra020870	*AT4G21810*	Der1-like family	Leaf
Bra020871	*AT4G21800*	Conserved hypothetical ATP binding protein; 5 0S ribosome-binding GTPase; Ras family	Leaf
Bra020872	*AT4G21750*	START domain; Homeobox domain	Flower
Bra020873	*AT4G21710*	RNA polymerase Rpb2, domain 2/3/4/5/6/7; RNA polymerase beta subunit; B3 DNA bindin g domain	Root
Bra020874	—	B3 DNA binding domain	—
Bra020875	*AT4G20930*	NAD binding domain of 6-phosphogluconate d ehydrogenase	Flower
Bra020876	*AT4G20940*	Leucine Rich repeats (2 copies)	Flower
