# Supplementary material for: Identification of Clubroot (Plasmodiophora brassicae) Resistance Loci in Chinese Cabbage (Brassica rapa ssp. pekinensis) with Recessive Character
Source: Genes (Basel). 2024 Feb 22;15(3):274. doi: 10.3390/genes15030274 (PMC10970103; doi:10.3390/genes15030274)
Supplement: Supplementary file 1 [file genes-15-00274-s001.zip › Table S5.pdf]

Table S5. Candidate genes information of *Cr4Ba1.1*

Gene ID	Homologous gene	Annotation	*A. thaliana* Highest expression
Bra013275	*AT4G18010*	Encodes an inositol polyphosphate 5-phosphatase, overexpression of this gene renders plants insensitive to ABA in germination and growth assay	Root
Bra013281	*AT1G11130*	Signal transduction mechanisms; Protein kinase/tyrosine domain	root
Bra013289	*AT4G18170*	WRKY DNA -binding domain	Root, flower
Bra013299	*AT4G18360*	Energy production and conversion; FMN-dependent dehydrogenase; Nitronate monooxygenase; IMP dehydrogenase / GMP reductase domain; Thiazole biosynthesis protein ThiG	Root
Bra013310	*AT4G18450*	AP2 domain; Ethylene-responsive transcription factor	—
Bra013336	*AT4G18640*	Protein tyrosine/ kinase kinase; Leucine rich repeat N-terminal domain; Leucine Rich repeats (2 copies)	Stem
Bra013339	*AT4G18670*	Leucine rich repeat; Leucine Rich repeats (2 copies); Leucine rich repeat N-terminal domain;	Flower
Bra013341	*AT4G18710*	Carbohydrate transport and metabolism	Root, stem
Bra013345	*AT4G18760*	Leucine Rich repeats (2 copies); Leucine rich repeat	Flower
Bra013347	—	F-box associated	—
Bra013348	—	F-box domain	—
Bra013357	*AT4G19920*	TIR domain	Root
