# Supplementary material for: Identification of Clubroot (Plasmodiophora brassicae) Resistance Loci in Chinese Cabbage (Brassica rapa ssp. pekinensis) with Recessive Character
Source: Genes (Basel). 2024 Feb 22;15(3):274. doi: 10.3390/genes15030274 (PMC10970103; doi:10.3390/genes15030274)
Supplement: Supplementary file 1 [file genes-15-00274-s001.zip › Table S6.pdf]

Table S6. The special primers sequences of candidate genes in A01 used in qRT-PCR

Gene	Primer	Primer sequence
Bra013281	3281-1	F: GAATTGCTCACTGGACGC<br>R: TCTCGTCAACGCATCTATG
Bra013289	3289-1	F: CGGAGATTAGTCGGAAGC<br>R: GCAAAGGTGCAATGTGAAG
Bra013336	3336-2	F: TCTTTACAGCTCTTTTACGGC<br>R: TTGCTTCTTTCCTCGGTT
Bra013339	3339-1	F: TGGGCGATCAGTTACTCCT<br>R: ATCATCGGAGGACTAGCTGG
Bra013341	3341-1	F: GTTCGATAGATCCATATTCGTC<br>R: ACTACCTCCAGAACCGGA
Bra013357	3357-3	F: CACCGTGAAACATCTCCG<br>R: GCCTCCTTCCATCTATTGG
Actin	Actin	F: GGAGCTGAGAGATTCCGTTG<br>R: GGAGCTGAGAGATTCCGTTG
